# Response to Rotenone Is Glucose-Sensitive in a Model of Human Acute Lymphoblastic Leukemia: Involvement of Oxidative Stress Mechanism, DJ-1, Parkin, and PINK-1 Proteins

**DOI:** 10.1155/2014/457154

**Published:** 2014-05-11

**Authors:** Miguel Mendivil-Perez, Marlene Jimenez-Del-Rio, Carlos Velez-Pardo

**Affiliations:** Neuroscience Research Group, Medical Research Institute, Faculty of Medicine, University of Antioquia (UdeA), Calle 70 No. 52-21 and Calle 62 No. 52-59, Building 1, Room 412, Medellin, Colombia

## Abstract

To establish the effect of low (11 mM) and high (55 mM) glucose concentrations (G11, G55) on Jurkat cells exposed to rotenone (ROT, a class 5 mitocan). We demonstrated that ROT induces apoptosis in Jurkat cells cultured in G11 by oxidative stress (OS) mechanism involving the generation of anion superoxide radical (O_2_^∙−^, 68%)/hydrogen peroxide (H_2_O_2_, 54%), activation of NF-*κ*B (32%), p53 (25%), c-Jun (17%) transcription factors, and caspase-3 (28%), apoptosis-inducing factor (AIF, 36%) nuclei translocation, c-Jun N-terminal kinase (JNK) activation, and loss of mitochondria transmembrane potential (ΔΨ_m_, 62%) leading to nuclei fragmentation (~10% and ~40% stage I-II fragmented nuclei, resp.). ROT induces massive cytoplasmic aggregates of DJ-1 (93%), and upregulation of Parkin compared to untreated cells, but no effect on PINK-1 protein was observed. Cell death marker detection and DJ-1 and Parkin expression were significantly reduced when cells were cultured in G55 plus ROT. Remarkably, metformin sensitized Jurkat cells against ROT in G55. Our results indicate that a high-glucose milieu promotes resistance against ROT/H_2_O_2_-induced apoptosis in Jurkat cells. Our data suggest that combined therapy by using mitochondria-targeted damaging compounds and regulation of glucose (e.g., metformin) can efficiently terminate leukemia cells via apoptosis in hyperglycemic conditions.

## 1. Introduction

Acute lymphoblastic leukemia (ALL) is a type of hematologic disorder characterized by excess production of lymphoblast cells. ALL represents 12% of all leukemia cases, with a worldwide incidence projected to 1–4.75 per 100,000 people [1]. Despite significant improvements in the treatment of ALL, pediatric (~30%) and adult (50–70%) patients develop treatment-resistant disease. Furthermore, hyperglycemia during hyper-CVAD (fractionated cyclophosphamide, vincristine, doxorubicin, and dexamethasone) chemotherapy is associated with poor outcomes of ALL in about 35% of patients [2]. However, whether the action of new therapeutic compounds is different in hyperglycemic conditions versus normoglycemia in ALL patients is still unknown. Because leukemia cells are metabolically flexible, this condition might potentiate ALL cells to develop treatment resistance. At present, evasion of apoptosis—a type of programmed cell death—is one of the eight essential alterations in cellular physiology leading to malignancy [3]. Apoptosis displays morphological and biochemical hallmarks [4, 5] involving chromatin condensation, nuclear fragmentation, rounding-up of the cell, reduction of cellular volume, and metabolic and energetic modifications of the mitochondria. Reactivation of apoptosis therefore appears as a prime strategy to eliminate cancer cells. Specifically, the identification of molecular pathways of cell death in ALL is an essential step towards therapeutic strategies. Before reaching this aim, the mechanism(s) must be unravelled.

Since mitochondria play essential roles in cellular metabolism, redox homeostasis, and the regulation of cell death, they represent an important target for anticancer therapy [6]. Interestingly, several natural compounds have been demonstrated to preferentially kill cancer cells with mitochondrial dysfunction [7]. Indeed, rotenone (ROT), a class 5 mitocan [8] that derives from the roots and backs of the* Derris* and* Lonchorcarpus* plant species, irreversibly binds to the* Complex I* (NADH: ubiquinone oxidoreductase) of the mitochondrial electron transport chain. This inhibition allows the reduction of molecular oxygen (O_2_) to superoxide anion radical (O_2_^∙−^) by the iron-sulfur cluster N2, and subsequent generation of H_2_O_2_, most probably by the flavin mononucleotide (FMNH^−^) of* Complex I* [9]. ROT induces apoptosis in several cancerous cells [10–13]. However, whether the activation of executor protease caspase-3 [13, 14] and the stress response c-Jun N-terminal protein kinase (JNK) pathway [12, 15, 16] occur in cancer cell lines exposed to ROT is not yet fully resolved. Moreover, ROT may [17] or may not [15] provoke cell death in pheochromocytoma PC12 cells. Therefore, the mechanism underlying ROT-induced apoptosis in cancer cells is not completely clear.

Recently, our group has provided evidence that oxidative stress (OS) generated by glucose-starvation (GS) induces apoptosis-inducing factor (AIF)- and caspase-3-dependent mitochondrial mechanisms of cell death in Jurkat cells (a model of human acute lymphoblastic leukemia) characterized by the activation of transcription factors such as nuclear factor-kappa B (NF-*κ*B), p53, c-Jun; activation of c-Jun N-terminal* kinase*, AIF translocation to nuclei, mitochondria depolarization, and nuclear fragmentation as evidence of caspase-3 activation [18]. These observations have led us to postulate aminimal completeness of cell death signaling induced by OS as a mechanistic explanation of cancer cell demise [19]. However, whether ROT induces a similar cell death pathway in Jurkat cells and whether ROT action can be regulated by glucose are not yet clear.

Interestingly, Zhang et al. [20] have shown that p53 increases the transcription of* PARKIN* gene both* in vitro* and* in vivo *under OS. In turn, Parkin contributes to the role of p53 in regulating glucose metabolism and antioxidant defense by activating NF-*κ*B [21, 22]. Surprisingly*, PARKIN* and other genes such as* PINK-1* (Phosphatase and tensin homolog (PTEN)-induced novel kinase-1) and *DJ*-1 have been associated with leukemia [23, 24]. How these molecules interplay with p53, NF-*κ*B, and AIF in OS-induced apoptosis in Jurkat cells under different metabolic conditions (e.g., glucose) is not yet established.

On the assumption that a high-glucose milieu may interfere with the cell response to drugs, we investigated the ROT-induced toxicity in low (11 mM, G11) and high (55 mM, G55) glucose concentrations that mimic* in vivo* conditions of normoglycemia and hyperglycemia, respectively. To get insight, we sought (i) to investigate whether ROT induces apoptosis in Jurkat cell line; (ii) to determine whether ROT treatment induces OS through O_2_^∙−^/H_2_O_2_, caspase-3, AIF, and the activation of proapoptotic transcription factors NF-*κ*B, p53, and c-Jun. We also investigated (iii) whether ROT differentially alters DJ-1, Parkin, and PINK-1 protein expression in Jurkat cells in both glucose conditions. The present investigation suggests that combined therapy by using mitocan compounds and metformin might efficiently terminate leukemia cells via apoptosis in hyperglycemic ALL patients.

## 2. Materials and Methods

3,3′-dihexyloxacarbocyanine iodide (D_i_OC_6_(3), Catalog number D-273), ammonium pyrrolidinedithiocarbamate (PDTC, Catalog number 548000), and 1,9-pyrazoloanthrone (SP600125, Catalog number 420119) were acquired from Calbiochem. Dichlorofluorescein diacetate (DCFH_2_-DA) was obtained from Invitrogen. All other reagents were purchased from Sigma-Aldrich.

### 2.1. Jurkat T Leukemia Cell Culture

Jurkat clone E6-1 (ATCC Catalog number TIB-152) was cultured according to supplier's indications.

### 2.2. Experiments with Jurkat Leukemia Cell Line

#### 2.2.1. Morphological Assessment of Cell Death by Fluorescent Microscopy

The cell suspension (1 mL, final volume) was exposed to increasing ROT (0–100 *μ*M) concentrations freshly prepared in RPMI-1640 medium either supplemented with 11 mM (hereafter 11G) or 55 mM (hereafter 55G) glucose in the absence or presence of different products of interest for 1–24 h at 37°C. Cells were incubated with 1 *μ*L acridine orange (AO, 0.1 mg/mL)/ethidium bromide (EB, 0.1 mg/mL)/Hoechst (H, 3 *μ*g/mL) staining. Quantification of apoptotic morphology was performed by fluorescent microscopy analysis. The apoptotic indexes were assessed 3 times in independent experiments blind to experimenter.

#### 2.2.2. Evaluation of Intracellular Reactive Oxygen Species (ROS)

Superoxide anion radicals were evaluated as described in Ref Quantification of nitroblue tetrazolium positive cells (NBT^+^) was performed blind to experimenter. The assessment was repeated 3 times in independent experiments. Hydrogen peroxide (H_2_O_2_) was determined with 2′,7′-dichlorofluorescin diacetate (5 *μ*M), using a flow cytometer Beckman Coulter Epics XL and fluorescent microscopy analysis, respectively. The assessment was repeated 3 times in independent experiments.

#### 2.2.3. Analysis of Mitochondrial Membrane Potential (ΔΨ_*m*_) by Flow Cytometry and Fluorescent Microscopy

Jurkat cell line was treated as described above. Then, cells (1 × 10^5^) were incubated with cationic lipophilic (10 nM) and (1 *μ*M) DiOC_6_(3) final concentration for 20 min at RT in the dark. Cells were then analyzed using flow cytometer Beckman Coulter Epics XL and fluorescent microscopy analysis, respectively. The assessment was repeated 3 times in independent experiments.

### 2.3. Immunocytochemistry Detection of Transcription Factor NF-*κ*B, p53, c-Jun, Caspase-3, Apoptosis-Inducing Factor (AIF), DJ-1, Parkin, and PINK-1

The Santa Cruz Biotechnology (SCB) supplier protocol goat ABC staining System (Catalog number sc-2023) was followed for the immunocytochemistry using primary goat polyclonal antibodies NF-*κ*B p65 (C-20)-G (Catalog number sc-372-G), p53 (FL-393) (Catalog number sc-6243-G), p-(Ser73)-c-Jun (Catalog number sc-7981), caspase-3 (Catalog number sc-22171) and AIF (Catalog number sc-9417) and SCB protocol goat ABC staining System (Catalog number SC-2018) was followed for the immunocytochemistry using primary antibodies DJ-1 (FL189) (Catalog number sc-32874), Parkin (H300) (Catalog number sc-30130) and PINK-1 (H300) (Catalog number sc-33796). The cells were immune-stained and diaminobenzidine positive (DAB^+^) cells were quantified blind to experimenter.

### 2.4. Detection of DJ-1, Parkin, and PINK-1 by Flow Cytometry

After each treatment with or without ROT in G11 or G55, cells (1 × 10^5^) were fixed in 80% ethanol and stored at −20°C overnight. Then after that, cells were washed with PBS and permeabilized with 0.2% triton X-100 plus 1.5% bovine serum albumin (BSA) in phosphate buffer solution (PBS) for 30 min. Cells were washed and incubated with 20 *μ*g/mL (diluted in PBS containing 0.1% BSA) primary antibody DJ-1 (FL189) (Catalog number sc-32874), Parkin (H300) (Catalog number sc-30130), and PINK-1 (H300) (Catalog number sc-33796) for 2 h at 37°C under agitation. Subsequently, the cells were washed and incubated with FITC-conjugated rabbit-antigoat-IgG antibody (Catalog number sc-2012; 20 *μ*g/mL) for 30 min at RT in the dark. After washing with PBS, the cells were resuspended in 500 *μ*L of PBS. Analysis was performed on a Flow Cytometer Beckman Coulter Epics XL. Jurkat cells without primary antibodies served as negative control. Results of the mean fluorescence intensity (MFI) of Jurkat cells were compared between controls and treatments. For assessment purpose, it was acquired 10,000 events and assessment was performed 3 times in independent experiments. Quantitative data were obtained using Windows Multiple Document Interface for Flow Cytometry 2.8 (WinMDI 2.8) software (http://facs.scripps.edu/software.html).

### 2.5. Evaluation of Signal Inhibitors on Cells Exposed to Rotenone

The cell suspension (1 mL, final volume) was treated with (50 *μ*M) ROT in glucose (11G) and inhibitor reagents at the concentration listed in Table 1 at 37°C for 24 h. After this time, cells were evaluated for apoptotic features (nuclei morphology and ΔΨ_m_) by fluorescence microscopy or flow cytometry, as described in previous sections. The assessment was repeated 3 times in independent experiments.

### 2.6. Statistical Analysis

Data are means ± S.D. of three independent experiments. One-way ANOVA analyses with Bonferroni or Games-Howell* post hoc* comparison were calculated with SPSS 18 software. A *P* value of *P < 0.05 and **P < 0.001 was considered significant.

### 2.7. Photomicrography

The light microscopy or fluorescent photomicrographs were taken using a Zeiss (Axiostart 50) microscope equipped with a Canon PowerShot G5 digital camera.

## 3. Results

### 3.1. Rotenone (ROT) Induces Nuclei Morphology Distinctive of Apoptosis in Jurkat T Cells Associated with Superoxide Anion Radical (O_2_^∙−^)/Hydrogen Peroxide (H_2_O_2_) Generation and Impairment of Mitochondrial Membrane Potential (ΔΨ_*m*_)

**Figure 1:**
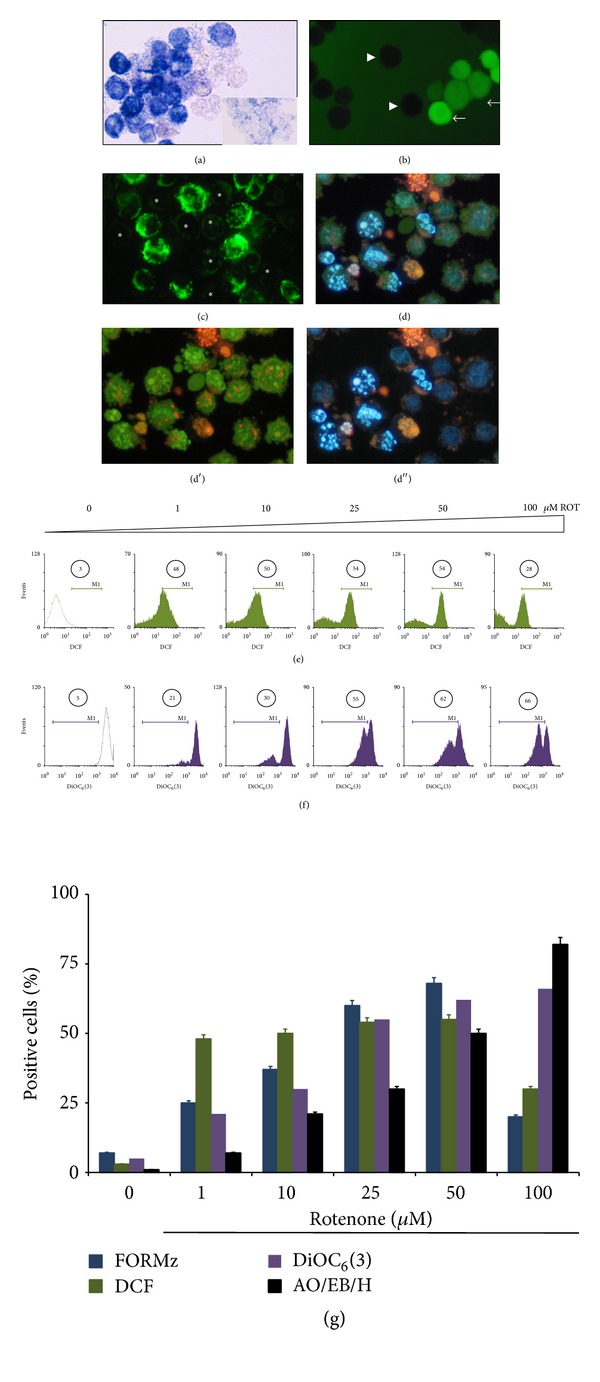
Rotenone (ROT) induces reactive oxygen species, mitochondrial depolarization, and chromatin condensation/nuclei fragmentation in Jurkat T leukemia cells. (a) Representative light photomicrography showing positive nitroblue tetrazolium (NBT^+^) stained blue-purple precipitate cells (i.e., formazan: FORMz) as positive O_2_^∙−^ generation in Jurkat cells cultured in basal glucose medium containing 11 mM glucose (G11) treated with (50 *μ*M) ROT for 24 h.* Inset*: untreated cells showing negative NBT staining. (b) Fluorescent photomicrography (ex. 450–490 nm, em. 515 nm) illustrating positive 2′,7′-dichlorofluorescein (DCF,* arrows*) and negative (*arrowheads*) stained cells as positive/negative H_2_O_2_ production from Jurkat T cells cultured in G11 treated with (50 *μ*M) ROT for 24 h. (c) Representative fluorescent photomicrography (ex. 450–490 nm, em. 515 nm) illustrates positive green fluorescent stained cells as DiOC_6_(3) high-polarized and low-polarized mitochondria and depolarized mitochondria (*asterisk*) from treated cells with (50 *μ*M) ROT in G11 for 24 h. (d) Representative (merge image) fluorescent photomicrography showing treated cells with (50 *μ*M ) ROT in G11 for 24 h with condensed chromatin (stage I nuclei morphology) and DNA fragmentation (stage II nuclei morphology) analyzed by either AO/EB ((d′), ex. 450–490 nm, em. 515 nm), Hoechst staining ((d′′), ex. 354 nm, em. 442 nm). (e) Representative histograms showing the percentage of dichlorofluorescein positive (DFC^+^) cells. (f) Percentage of DiOC_6_(3) low (DiOC_6_(3)^−^) cells exposed to increasing concentration of ROT (0–100 *μ*M), assessed at 24 h. (g) The percentage of positive FORMz, DCF, DiOC_6_(3), and AO/EB/Hoechst cells exposed to increasing concentration of ROT (0–100 *μ*M) is expressed as a mean percentage ± S.D. from three independent experiments. Magnification ((a)–(d)) 1000x. Magnification inset (a) 600x.

We initially evaluated the effect of ROT in Jurkat cells under standard culture conditions (glucose medium containing 11 mM glucose, G11). As shown in Figure 1, ROT induced generation of O_2_^∙−^ (Figure 1(a)) in a concentration-dependent fashion up to 50 *μ*M (from 25% to 58%), declining to ~20% FORMz^+^ at 100 *μ*M (Figure 1(g)). On the contrary, ROT generated H_2_O_2_ (Figure 1(b)) almost constantly (48–55% DCF^+^) up to 50 *μ*M, but its generation was reduced by ~30% at 100 *μ*M (Figures 1(e), 1(g)). Concurrently, ROT provoked loss of ΔΨ_m_ (Figures 1(c) and 1(f)) and nuclei condensation/fragmentation (Figure 1(d)) in Jurkat cells in a concentration fashion (Figures 1(f) and 1(g)), as assessed by fluorescence microscopy (Figures 1(c) and 1(d)) and flow cytometry (Figures 1(f), 1(g), and 6(a)), respectively. Because 50 *μ*M ROT provoked ~50% nuclei fragmentation/ΔΨ_m_ depolarization and it is an end-point for O_2_^∙−^/H_2_O_2_ generation, this concentration was selected for further experiments. Of note, the antioxidant* N-acetyl-cysteine* (NAC, 1 mM) significantly reduced the proapoptotic effect of ROT in Jurkat cells (Table 1).

### 3.2. ROT Induces Apoptosis in Jurkat T Cells Associated with NF-*κ*B, p53 and c-Jun Transcription Factors, Caspase-3 Activation, and Apoptosis-Inducing Factor (AIF)

**Figure 2:**
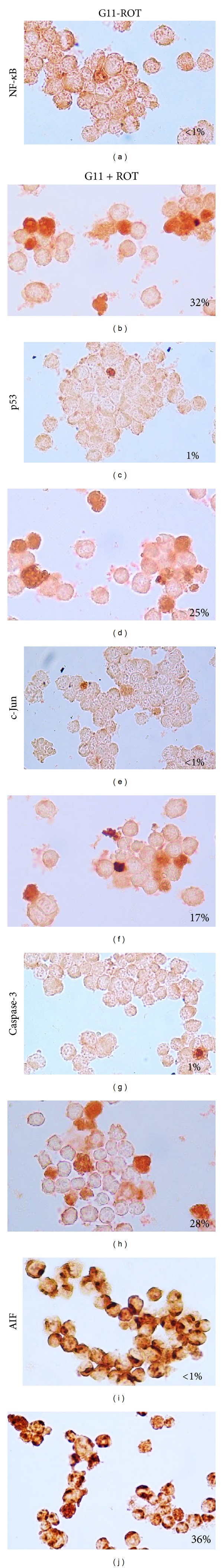
Rotenone induces activation of the transcription factors, apoptosis-inducing factor, and caspase-3 in Jurkat T cells. Leukemia cells were left untreated ((a), (c), (e), (g), and (i)) or exposed to (50 *μ*M) ROT ((b), (d), (f), (h), and (j)) in G11 medium for 24 h. Cells were stained with anti-NF-*κ*B-p65 ((a) and (b)), anti-p53 ((c) and (d)), anti-c-Jun ((e) and (f)), anti-caspase-3 ((g) and (h)), and anti-AIF ((i) and (j)), antibodies according to procedure described in* Materials and Methods*. Notice that positive nuclei (dark brown color) reflect their nuclear translocation/activation and appear to correlate with the apoptotic nuclear morphology. Magnification 1000x ((a)–(j)).* Insets*: percentage of positive DAB staining.

To evaluate the participation of signaling molecules in ROT-induced apoptosis, Jurkat cells were either preincubated one hour with PDTC (10 nM, an inhibitor of NF-*κ*B), PFT (50 nM, a specific inhibitor of p53), SP600125 (1 *μ*M, a specific inhibitor of c-Jun N-terminal kinase), or NSCI (10 *μ*M, a specific inhibitor of caspase-3) prior to incubation with ROT (50 *μ*M) in G11 for 24 h. All the specific pharmacological inhibitors moderately reduced the ROT-induced apoptotic effect and the ΔΨ_m_ compared to control (Table 1). The participation of the transcription factors, caspase-3, and AIF was also evaluated by immunohistochemistry. As shown in Figure 2, ROT clearly induced DAB^+^ nuclei staining of the active form of NF-*κ*B (Figure 2(b)), p53 (Figure 2(d)), c-Jun (Figure 2(f)), CASP-3 (Figure 2(h)), and AIF (Figure 2(j)) compared to untreated cells (Figures 2(a), 2(c), 2(e), 2(g), and 2(i)) with a different percentage of detection (Figure 2, insets). Noticeably, the morphology of the stained DAB^+^ cells corresponded exactly to the nuclei fragmentation observed under fluorescent microscopy (see Figures 1(d) and 2(b), 2(d), 2(f), 2(h), and 2(j)).

### 3.3. ROT Induces DJ-1 Cytoplasmic Accumulation and Parkin Overexpression but Has No Influence on PINK-1 Protein

**Figure 3:**
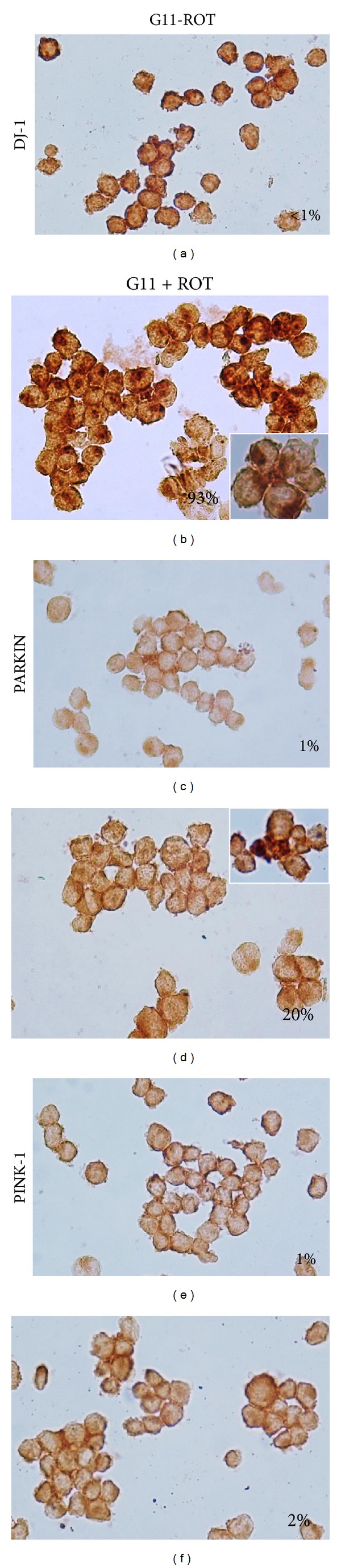
Rotenone induces differential effect on DJ-1, Parkin, and PINK-1 proteins (chromofore immunohistochemistry assay). Leukemia cells were left untreated ((a), (c), and (e)) or exposed to (50 *μ*M) ROT ((b), (d), and (f)) in G11 medium for 24 h. Cells were stained with anti-DJ-1 ((a) and (b)), anti-Parkin ((c) and (d)) and anti-PINK-1 ((e) and (f)) antibodies, as described in* Materials and Methods*. Notice that positive aggregates (dark brown color) reflect their cytoplasmic localization. Magnification 800x ((a)–(f)).* Insets*: percentage of positive DAB staining.

**Figure 4:**
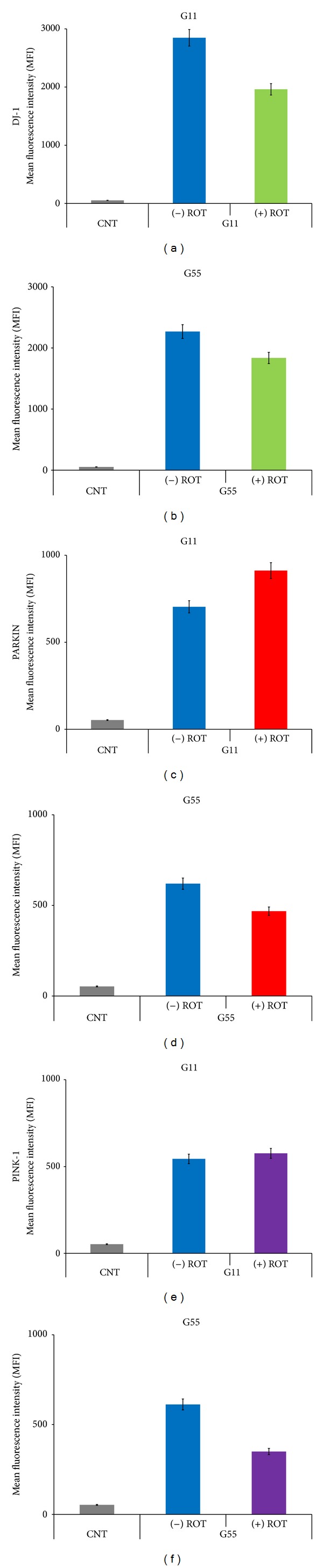
Glucose modulates the response of DJ-1, Parkin, and PINK-1 in Jurkat cells exposed to ROT. Leukemia cells were left untreated or exposed to (50 *μ*M) ROT in G11 or G55 medium for 24 h. Cells were stained with anti-DJ-1 ((a) and (b)), anti-Parkin ((c) and (d)), and anti-PINK-1 ((e) and (f)) antibodies, as described in* Materials and Methods*. Results are expressed as mean fluorescence intensity (MFI) of Jurkat cells and were compared between treatments from three independent experiments. *P < 0.05 versus respective control determined by the Student *t*-test.

Next, we examined whether DJ-1, Parkin, and PINK-1 were involved in ROT-induced cell death. As shown in Figure 3, ROT provoked massive cytoplasmic DAB^+^ aggregates of DJ-1 (93%, Figure 3(b)) compared to untreated cells (1%, Figure 3(a)), whereas Parkin and PINK-1 presented moderate or scarce DAB^+^ aggregates, respectively (Figures 3(d) and 3(f)) compared to untreated cells (Figures 3(c) and 3(e)), as assessed by immunocytochemistry. These observations were further confirmed by flow immunocytometry (Figures 4(a), 4(c), and 4(d)). Indeed, cells exposed to ROT showed low DJ-1 mean fluorescence intensity (MFI) units, reflecting cytoplasmic high molecular aggregates, compared to untreated cells (Figure 4(a)). In contrast, cells exposed to ROT showed higher Parkin MFI, reflecting either overexpression or the presence of Parkin monomers rather than aggregates, compared to untreated cells (Figure 4(c)). PINK-1 MFI protein levels were unaffected in cells under ROT exposure (Figure 4(e)).

### 3.4. High Glucose Reduces ROT-Induced Apoptosis Markers in Jurkat T Cells

**Figure 5:**
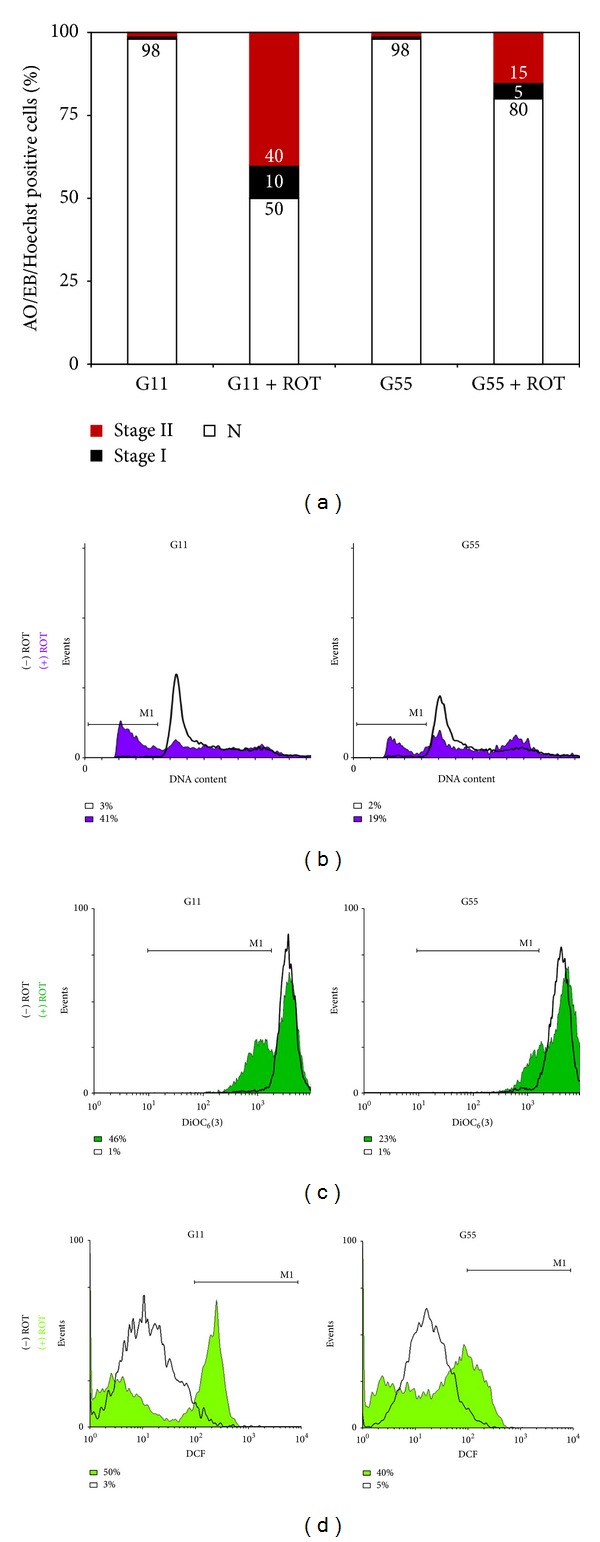
High glucose modulates ROT-induced apoptosis in Jurkat cells. Jurkat cells were left untreated (*white box*) or treated with (50 *μ*M) ROT (*purple, dark green, and light green box*) in G11 or G55 medium for 24 h. Then after that, cells were evaluated for (a) morphological changes (i.e., normal (*white box*), Stage I (*black box*) and II (*red box*) nuclei morphology) by fluorescence microscopy using AO/EB/Hoechst staining, (b) DNA fragmentation, (c) mitochondrial transmembrane potential, and (d) H_2_O_2_ generation, as described in* Materials and Methods.* Figures represent one out of three independent experiments andparameter values are expressed as percentage (%).

**Figure 6:**
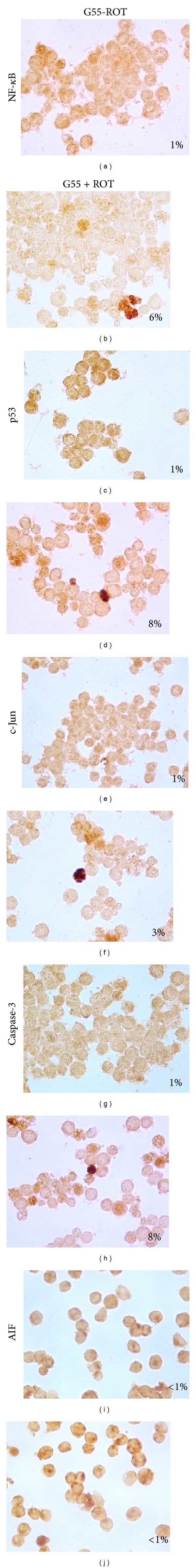
High glucose reduces the activation of the transcription factors, apoptosis-inducing factor, and caspase-3 in Jurkat T cells exposed to ROT. Leukemia cells were left untreated ((a), (c), (e), (g), and (i)) or exposed to (50 *μ*M) ROT ((b), (d), (f), (h), and (j)) in G55 medium for 24 h. Cells were stained with anti-NF-*κ*B-p65 ((a) and (b)), anti-p53 ((c) and (d)), anti-c-Jun ((e) and (f)), anti-caspase-3 ((g) and (h)), and anti-AIF ((i) and (j)) antibodies, as described in* Materials and Methods*. Notice that positive nuclei (dark brown color) reflect their nuclear translocation/activation and appear to correlate with the apoptotic nuclear morphology. Magnification 500x ((a)–(j)).* Insets*: percentage of positive DAB staining.

**Figure 7:**
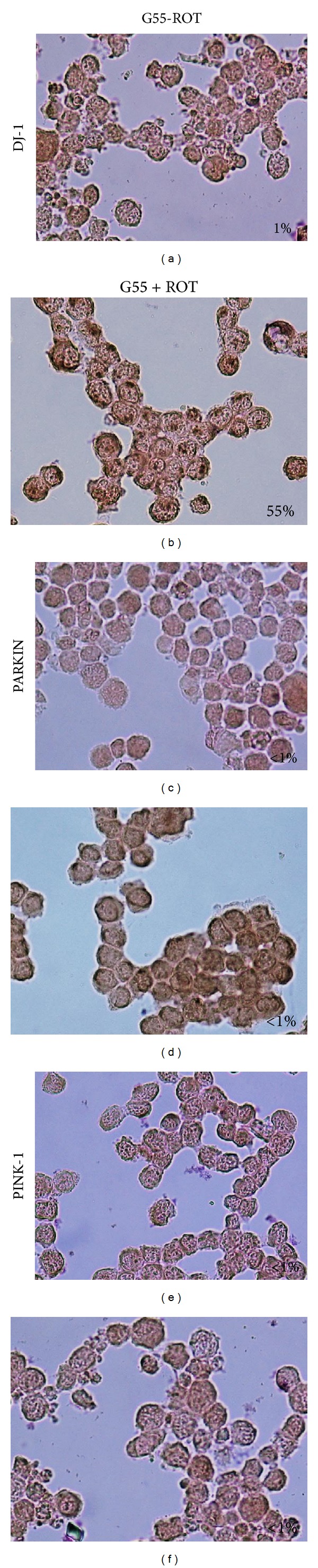
High glucose reduces DJ-1 aggregation in Jurkat T cells exposed to ROT. Leukemia cells were left untreated ((a), (c), and (e)) or exposed to 50 *μ*M ROT ((b), (d), and (f)) in G55 medium for 24 h. Cells were stained with anti-DJ-1 ((a) and (b)), anti-Parkin ((c) and (d)), and anti-PINK-1 ((e) and (f)) antibodies, as described in* Materials and Methods*. Notice that positive aggregates (dark brown color) reflect their cytoplasmic localization. Magnification 800x ((a)–(f)).* Insets*: percentage of positive DAB staining.

Since glucose metabolism is upregulated in cancer cells, we were interested in determining the extent to which glucose modulates ROT-induced apoptosis response in Jurkat cells. We characterized chromatin condensation as stage I nuclei morphology composed of high molecular weight DNA and nuclear fragmentation as stage II nuclei morphology composed of low molecular weight DNA, chromatin condensation in highly packed round masses of nuclei morphology induced by (50 *μ*M) ROT under (55 mM) glucose concentration (i.e., G55). As shown in Figure 5(a), ROT induced lower apoptotic nuclei morphology (20%) with a different stage I (~5%) and stage II (~15%) nuclei morphology proportion under G55 than in cells cultured under G11 plus ROT (50% apoptotic nuclei: 10% stage I, 40% stage II nuclei morphology), as evaluated by triple AO/EB and Hoechst staining techniques (Figure 1(d)). These observations were confirmed by DNA fragmentation (Figure 5(b)) and ΔΨ_m_ depolarization (Figure 5(c)), as evaluated by flow cytometer. Of note, the ΔΨ_m_ was significantly preserved in cells exposed to ROT in G55 compared with cells exposed to ROT in G11. We then established generation of ROS, activation of signaling (NF-*κ*B, p53), executer (caspase-3, AIF), and mitochondrial maintenance (DJ-1, Parkin, and PINK-1) molecules under G55 condition. As noted in Figure 5(d), cells exposed to ROT generated high levels of H_2_O_2_, shown as MFI, irrespective of glucose medium concentration. Likewise, cells under G55 + ROT showed a low percentage of NF-*κ*B (Figure 6(b)), p53 (Figure 6(d)), c-Jun (Figure 6(f)), caspase-3 (Figure 6(h)) activation, and AIF (Figure 6(j)) compared with cells under G55 alone (Figures 6(a), 6(c), 6(e), 6(g), and 6(i)). Furthermore, cells exposed to G55 plus ROT also displayed low percentage of DJ-1 (Figures 4(b) and 7(b)) and Parkin (Figures 4(d) and 7(d)), evaluated as DAB^+^ cells and MFI units, respectively. PINK-1 showed similar percentages of DAB^+^ cells and MFI units in cells under G55 and ROT (Figures 4(f) and 7(f)).

### 3.5. Metformin Sensitizes Jurkat Cells against ROT-Induced Toxicity

**Figure 8:**
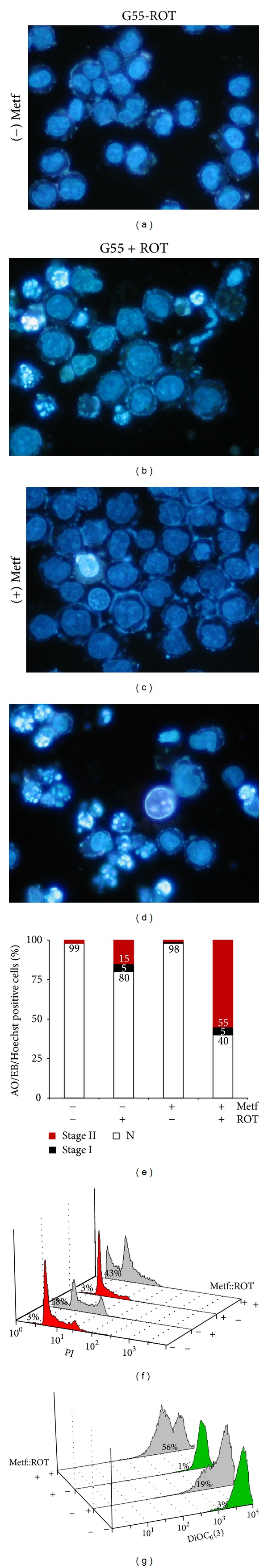
Metformin sensitizes Jurkat cells against ROT exposure in high glucose (G55) condition. Leukemia cells were left untreated ((−) Metf, (−) ROT), exposed to (+) ROT (50 *μ*M) alone or in combination of metformin in G55 medium for 24 h. Then after that, cells were evaluated ((a)–(d)) and (e) quantified for morphological changes (i.e., Stage I and II nuclei morphology) by fluorescence microscopy using AO/EB/Hoechst staining, (f) DNA fragmentation assay. (g) Mitochondrial transmembrane potential assay, as described in* Materials and Methods.* The values in (e) are expressed as mean from three independent experiments. Magnification 1000x ((a)–(e)).

Since metformin, a recognized glucose-lowering drug, has been shown to strongly interfere with survival and proliferation on Jurkat cells [25], we wanted to know whether metformin affected the toxicity of ROT in Jurkat cells under G55. As shown in Figure 8(e), metformin increases ~3-fold ROT-induced apoptosis in Jurkat cells in G55, as assessed by AO/EB/Hoechst staining (Figures 8(a)–8(d)), DNA fragmentation (Figure 8(f)), and ΔΨ_m_ depolarization assay (Figure 8(g)).

## 4. Discussion

Several data suggest that ROT induces death through enhancing O_2_^∙−^/H_2_O_2_ production by mitochondrial complex I [9] in cancer cells [13, 26]; however, the mechanism by which ROT provokes cell death remains controversial (i.e., OS versus the impairment of ΔΨ_m_ and decline of energy production). Here, we provide* in vitro* evidence supporting a role for OS in ROT-induced apoptosis in Jurkat cells under 2 different glucose (G) milieus: 11 mM (G11) and 55 mM (G55) glucose, as a model of normoglycemia and hyperglycemia in ALL, respectively. Mechanistically, ROT-induced apoptosis complies with the model of minimal completeness of cell death signaling [19]. Effectively, we confirm that ROT (1–100 *μ*M) produces O_2_^∙−^ and H_2_O_2_ in Jurkat cells according to the reduction of NBT to formazan and DFCH_2_ oxidation to fluorescent DCF assay, respectively. Furthermore, ROT generated O_2_^∙−^ in a concentration-dependent fashion up to 50 *μ*M with a marked decreased at 100 *μ*M; however, H_2_O_2_ was constantly produced up to 50 *μ*M with a significant reduction at 100 *μ*M. Why did O_2_^∙−^ and H_2_O_2_ levels decrease at 100 *μ*M ROT concentration compared with lower ROT concentrations? A possible explanation is that 100 *μ*M ROT is high enough concentration to generate high amounts of O_2_^∙−^, which can dismutate enzymatically or nonenzymatically into H_2_O_2_ at early ROT exposure with no apparent effect on nuclei morphology and ΔΨ_m_. Once produced, H_2_O_2_ triggers nuclei and ΔΨ_m_ damage. By 24 h, O_2_/O_2_^∙−^/H_2_O_2_ have been exhausted, destroyed, or decomposed concomitantly with maximal percentage of cell nuclei condensation/fragmentation and loss of ΔΨ_m_. Because 50 *μ*M ROT provoked ~50% nuclei fragmentation/ΔΨ_m_ depolarization, and it was an end-point for O_2_^∙−^/H_2_O_2_ generation, this concentration was selected for testing. Interestingly, 50 *μ*M ROT generates high amounts of ROS in G11 and G55. This observation implies that ROT produces O_2_^∙−^ and H_2_O_2_ independently of glucose concentration. However, the specific behavior of effector (NF-*κ*B, p53, c-Jun), executer (caspase-3, AIF), antioxidant (DJ-1), and mitochondrial related (Parkin, PINK-1) molecules changed according to milieu condition. We found that ROT induces chromatin condensation (stage I) and nuclei fragmentation (stage II) concomitantly with loss of ΔΨ_m_, nuclear localization of AIF, NF-*κ*B, p53, c-Jun, and caspase-3 markers and apoptotic nuclear morphology in G11. Furthermore, while ROT induced cytoplasmic DJ-1 protein accumulation and the upregulation of Parkin, PINK-1 protein kinase was unaffected. In contrast, when glucose was present in excess (G55), cell death and/or dysfunctional markers were dramatically (>50%) reduced. Taken together, our data suggest that ROT induces apoptosis in Jurkat cells more efficiently in low (G11) than in high (G55) glucose environment by OS molecular mechanism. Several data support this conclusion. First, ROT-induced apoptosis in Jurkat cells was significantly reduced by the antioxidant NAC [26]. Second, H_2_O_2_ was constantly produced by ROT exposure (up to 50 *μ*M for 24 h). Currently, H_2_O_2_ is considered an intracellular second messenger in several signalling pathways by activating kinases. Interestingly, some reports have demonstrated that H_2_O_2_ induces NF-*κ*B activation in Jurkat cells either through the spleen tyrosine kinase (Syk) [27] or the I*κ*B*α* kinase complex (IKK) [28]. Noticeably, Jurkat cells treated with ROT induced p65-DAB^+^ nuclei, as an indicator of p65 activation and translocation to the nuclei. Moreover, pharmacological inhibition of NF-*κ*B with PDTC significantly inhibited the apoptotic morphology under ROT exposure. These data suggest that ROT induces activation and translocation of the NF-*κ*B (p65) probably via the aforementioned H_2_O_2_-induced mechanisms, thus implicating the activation of the transcription factor NF-*κ*B in ROT-induced cell demise. In accordance with other scientific reports (e.g., [29]), our data suggest that NF-*κ*B functions as a sensor of OS linked to cell death signaling. Third, it has been shown that NF-*κ*B is able to upregulate p53 expression in cells exposed to H_2_O_2_ [30]. Accordingly, it is found that ROT induces p53 DAB^+^ cells with evident morphology of apoptotic nuclei. This observation implies p53 as an important molecule in ROT-induced apoptosis. This conclusion is further supported by the fact that PFT, a specific inhibitor of p53, was able to significantly reduce ROT-induced apoptotic morphology and ΔΨ_m_ depolarization. Our observations suggest an association between NF-*κ*B and p53 in Jurkat cells under OS. Finally, inhibition of JNK, reduced activation of c-Jun, and low percentage of cell death in presence of ROT indicates that c-Jun activation is also required for ROT-induced cell death [31]. Collectively, these data suggest that NF-*κ*B, p53, JNK, and c-Jun are critical proapoptotic factors in ROT-induced apoptosis in Jurkat cells.

Apoptosis is a morphological phenomenon as an outcome of the biochemical process taking place at the mitochondria [5]. To avoid potential confusion about the mode of cell death in Jurkat cells with other techniques as reported by Mattes [32], we used one of the most reliable methods to directly visualize apoptotic morphology—the acridine orange/ethidium bromide assay in combination with Hoechst staining. ROT-induced morphological apoptotic features (i.e., stage I and II nuclei fragmentation) in Jurkat cells. These observations suggest that ROT induces apoptosis via 2 pathways which run in parallel: AIF-dependent and caspase-3-dependent mechanisms associated with mitochondrial depolarization. This assumption is supported by a significant reduction of apoptosis in Jurkat cells treated with specific caspase-3 inhibitor NSCI, and caspase-3/AIF immunohistochemical positive nuclei (DAB^+^) staining. These results comply with the notion that caspase-3 is required for DNA fragmentation and some of the typical morphological changes of cells undergoing apoptosis such as shrinkage, blebbing, and the appearance of nuclei fragmentation in packed round masses, while AIF is required for chromatin fragmentation (for a review, see [33]). Remarkably, we observed neither cytoplasmic vacuolization, nor swelling of the cell, nor mild clumping of nuclear chromatin on cells treated with ROT. Our findings strongly suggest that Jurkat cells die by apoptosis rather than by other modes of cell death. In conclusion, ROT-induced apoptosis proceeds in a domino-like mechanism triggered by O_2_^∙−^/H_2_O_2_, which in turn activates (≫) kinases > NF-*κ*B, JNK (>c-Jun) > p53 ≫ ΔΨ_m_ > caspase-3, AIF > chromatin condensation (stage I), and nuclei fragmentation (stage II).

ROT induces massive cytoplasmic aggregation of DJ-1 in Jurkat cells cultured in G11 compared to untreated cells in either G11 or G55 milieu. These findings are in agreement with the notion that H_2_O_2_ may be able to modify the structural disposition of DJ-1 [34]. Indeed, it has been reported that H_2_O_2_ is able to oxidizing Cysteine_106_ residue into cysteine sulfonate (Cys_106_-SO_3_^−^), thereby provoking DJ-1 aggregates [35]. However, whether this oxidative modification has a functional role in DJ-1 is unknown. Despite this drawback, several data suggest that Cys_106_ is required for DJ-1 to confer cellular protection against OS [36]. Current evidence suggest that under basal conditions, DJ-1 (intact Cys_106_) might function as an antioxidant protein by either scavenging relatively low H_2_O_2_ concentrations or performing other prosurvival ancillary functions (e.g., as chaperone). Under moderate H_2_O_2_ exposure, oxidized DJ-1 (cysteine-sulfinate Cys_106_-SO_2_^−^) protein might function as an inhibitor of either proapoptotic proteins (e.g., apoptosis signal regulating kinase-1, ASK-1; p53) by preventing buildup of H_2_O_2_ in mitochondria [37] or of the upregulation of *γ*-glutamylcysteine ligase—the rate-limiting enzyme in the glutathione (GSH) biosynthetic pathway. This last assumption is compatible with our observation that NAC, an antioxidant and precursor of GSH, protects Jurkat cells against ROT. However, under extreme or constant H_2_O_2_ generation, such as by ROT, further oxidation of DJ-1 (cysteine-sulfonate Cys_106_-SO_3_^−^) elicits loss of structure and aggregation [34]. Because DJ-1 is a sensitive protein towards oxidation by H_2_O_2_, this protein may constitute a marker of cellular stress.

Accumulating evidence suggests that Parkin and PINK-1 may play a role in maintaining mitochondrial function and in preventing OS [38]. We found that ROT/H_2_O_2_ upregulates Parkin expression [39], most probably through p53 transcriptional regulation [20]. Our results indicate that ROT induces the differential expression of Parkin and PINK-1 proteins. What role does Parkin play in ROT-induced apoptosis? Because phosphorylation of Parkin by PINK-1 activates Parkin E3 ligase function, which in turnactivates NF-*κ*B signaling through the I*κ*B kinase/NF-*κ*B pathway [21, 22], we speculate that the PINK-1/Parkin interaction might amplify the NF-*κ*B/p53 death signal axis. Therefore, ROT induces a vicious cycle wherein H_2_O_2_ indirectly triggers PINK-1, which in turn activates (>) Parkin > NF-*κ*B > p53 > Parkin. Our findings suggest that PINK-1/Parkin might be important target proteins to accelerate cell death in Jurkat cells when treated with pro-oxidant drugs.

Recently, it has been shown that glucose starvation (GS) induces OS and apoptosis (~85%) through AIF- and caspase-3 dependent mechanisms [18]. Here, we found that excess glucose (G55) significantly reduces ROT-induced apoptosis (~20%) in Jurkat cells. Accordingly, the signaling death markers (NF-*κ*B, p53), executer (caspase-3, AIF), antioxidant (DJ-1), and mitochondria maintenance (Parkin, PINK-1) proteins diminished (>50%) compared to cells treated with ROT in G11. Our results suggest that high glucose promotes stress resistance against ROT/H_2_O_2_-induced apoptosis in Jurkat cells. How do cells manage to become resistant to ROT-induced apoptosis in G55? When cells are exposed to high OS stimuli, glucose can be routed through the pentose phosphate pathway (PPP) to generate the reducing agent NADPH, which preserves the intracellular levels of GSH, one of the most abundant intracellular antioxidant (for a review, see reference [40]). For this reason, cells with an active PPP are protected against OS and ROS-generating xenobiotics such as ROT. Therefore, alteration in energy metabolism by availability of glucose strictly correlated with degree of resistance to class 5 mitocan. However, whether high glucose affects the response of ALL cells to other mitocans (e.g., class 1–4, 6–8) [8] is still unknown. Further investigation is warranted to solve this issue. Interestingly, we found that the glucose-lowering drug metformin sensitizes Jurkat cells to ROT in G55 milieu. Grimaldi et al. [41] have found that metformin alone induced apoptosis and autophagy in ALL cells including Jurkat cells. Others have reported that metformin exerts its antidiabetic effects through inhibition of complex I of the mitochondrial respiratory chain [42]. However, we found that cells exposed to metformin alone were morphologically and metabolically normal, according to AO/EB/Hoechst staining, DNA fragmentation, and ΔΨ_m_ assay in both glucose conditions. Our data indicate therefore that the toxic effect of metformin may not involve inhibition of Complex I in Jurkat cells, at least under the present experimental conditions. Noticeably, metformin in presence of ROT in G55 dramatically induces apoptosis and nonapoptosis cell death. These data suggest that metformin in combination with Complex-I inhibitors might be a useful therapeutic strategy in hyperglycemic ALL patients [43].

**Figure 9:**
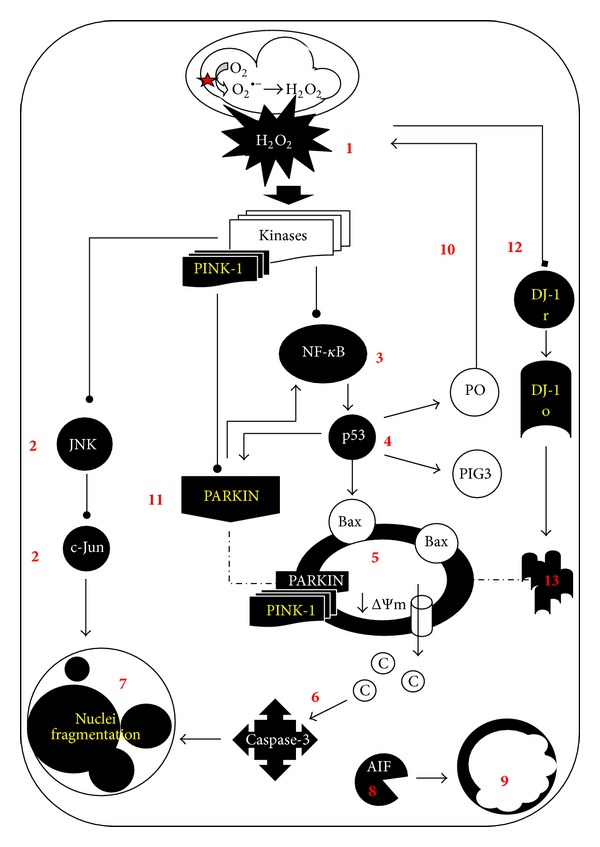
Schematic model of the major molecular events by which rotenone provokes apoptosis in Jurkat cells under different glucose conditions. Rotenone inhibits complex-I (NADH: ubiquinone oxidoreductase) ensuing overproduction of superoxide anion radicals (O_2_^∙−^) which dismutate enzymatically (by superoxide dismutase, SOD) or nonenzymatically into hydrogen peroxide (H_2_O_2_). This last compound freely diffuses through mitochondrial membranes to cellular cytoplasm (1). H_2_O_2_ may in turn activate one or several kinases (e.g., the mitogen-activated protein kinase kinase kinase 1, MEKK1; spleen tyrosine kinase, Syk; apoptosis signal regulating kinase-1, ASK) which can directly or indirectly activate both JNK/c-Jun (2) and NF-*κ*B (3) via phosphorylation of the I*κ*B*α* (i.e., the repressor of NF-*κ*B). Once NF-*κ*B is active, it translocates into the nucleus and transcribes p53 protein (4). In turn, p53 transcribes proapoptotic proteins (e.g., Bax) which are able to permeabilize mitochondria (5), thus promoting the activation of caspase-3 (6), which signals DNA fragmentation ((7), stage II nuclei fragmentation), and chromatin fragmentation ((9), stage I nuclei fragmentation) as a result of the apoptotic-inducer factor (AIF) protein action (8). Additionally, p53 can establish two independent but complementary vicious cycles. It can transcribe pro-oxidant proteins (e.g.,* p53-induced gene-3 (PIG3), proline oxidase (PO),* (10), which generate more H_2_O_2_ (1) amplifying the initial H_2_O_2_ > kinases > NF-*κ*B > p53 cell death signaling. p53 can also transcribe Parkin protein (11), which is phosphorylated by the autophosphorylated PINK-1 protein, to further activate NF-*κ*B (3), thus amplifying the NF-*κ*B > p53 > Parkin death axis. The overproduction of H_2_O_2_ also causes DJ-1 (12) oxidation (DJ-1 o) of Cysteine_106_ residue into cysteine sulfonate (Cys_106_-SO_3_^−^). This chemical reaction might change the structural conformation of DJ-1 into protein aggregates (13). However, when cells are cultured in high glucose concentration (e.g., 55 mM glucose) in presence of ROT, they are able to properly mount an antioxidant response (e.g., probably via pentose phosphate pathway (PPP) to increase GSH by NADPH generation). Hence, functional mitochondria and normal nuclei morphology are preserved.

In summary, we provide mechanistic evidence explaining the toxic effect of ROT in Jurkat cells under 2 different glucose conditions (Figure 9). ROT induces O_2_^∙−^/H_2_O_2_ generation, mitochondrial depolarization, NF-*κ*B, p53, and c-Jun transcription factor activation, AIF nuclear translocation, caspase-3 activation, chromatin condensation (stage I) and nuclei fragmentation (stage II), DJ-1 cytoplasmic aggregates, and upregulation of Parkin, typical characteristics of apoptosis in Jurkat cells cultured in G11 milieu. However, those apoptosis markers were significantly reduced in cells cultured in G55 under similar OS stimuli. Hyperglycemia in patients with leukemia is associated with increased hospital mortality. Here, we provide insight into the signaling and metabolic alterations related with drug response to metabolic milieu analogous to diabetic and nondiabetic situation in patients with ALL. Taken together, our data suggest that combined therapy by using mitochondrial targeted damaging compounds and regulation of glucose (e.g., by metformin) might efficiently terminate leukemia cells via apoptosis in hyperglycemic conditions.

## Figures and Tables

**Table 1:** Oxidative stress and signalling molecules (NF-*κ*B, c-Jun, p53, and caspase-3) are involved in rotenone (ROT)-induced apoptosis in Jurkat T cells.

Treatment/assay	AO/EB/H (%)	PI^−^/DiOC_6_(3)^+^ (%)
Untreated	1 ± 0**	98 ± 1**
ROT (50 *μ*M)	50 ± 3**	51 ± 2**
PDTC (10 nM)	1 ± 0**	97 ± 1**
PDTC (10 nM) + ROT (50 *μ*M)	9 ± 3**	89 ± 3**
PFT (50 nM)	1 ± 0**	98 ± 1**
PFT (50 nM) + ROT (50 *μ*M)	11 ± 2**	87 ± 3**
SP600125 (1 *μ*M)	2 ± 1**	97 ± 1**
SP600125 (1 *μ*M) + ROT (50 *μ*M)	6 ± 2**	93 ± 3**
NSCI (10 *μ*M)	1 ± 0**	98 ± 1**
NSCI (10 *μ*M) + ROT (50 *μ*M)	5 ± 1**	94 ± 2**
NAC (1 mM)	1 ± 0**	98 ± 1**
NAC (1 mM) + ROT (50 *μ*M)	10 ± 3**	89 ± 1**

Jurkat cells (1 × 10^6^ cells/mL) were exposed to ROT (50 *μ*M) in absence or presence of PDTC (10 nM), pifithrin-*α* (PFT, 50 nM), SP600125 (1 *μ*M) and NSCI (10 *μ*M) signaling inhibitors, and NAC (1 mM) at 37°C for 24 h. Cells were then evaluated for apoptotic nuclei morphology and mitochondrial membrane potential, as described in Material and Methods. The percentage of positive AO/EB/Hoechst staining and PI^−^/DiOC_6_(3)^+^ of Jurkat cells treated with or without ROT is expressed as a mean percentage ± S.D. from three independent experiments. **P < 0.001.
